# Physician experience with speech recognition software in psychiatry: usage and perspective

**DOI:** 10.1186/s13104-018-3790-y

**Published:** 2018-10-01

**Authors:** John Fernandes, Ian Brunton, Gillian Strudwick, Suman Banik, John Strauss

**Affiliations:** 1 Shannon Centennial Informatics Lab, Centre for Addiction and Mental Health, 1001 Queen St W, Toronto, M6J 1H4 Canada; 2 Women’s College Hospital, Toronto, Canada; 3 University of Toronto, Toronto, Canada

**Keywords:** Psychiatry documentation, Mental health, Speech recognition software, Behavioral health

## Abstract

**Objective:**

The purpose of this paper is to extend a previous study by evaluating the use of a speech recognition software in a clinical psychiatry milieu. Physicians (n = 55) at a psychiatric hospital participated in a limited implementation and were provided with training, licenses, and relevant devices. Post-implementation usage data was collected via the software. Additionally, a post-implementation survey was distributed 5 months after the technology was introduced.

**Results:**

In the first month, 45 out of 51 (88%) physicians were active users of the technology; however, after the full evaluation period only 53% were still active. The average active user minutes and the average active user lines dictated per month remained consistent throughout the evaluation. The use of speech recognition software within a psychiatric setting is of value to some physicians. Our results indicate a post-implementation reduction in adoption, with stable usage for physicians who remained active users. Future studies to identify characteristics of users and/or technology that contribute to ongoing use would be of value.

## Introduction

For a number of years, physicians have used speech recognition software (SRS) to support clinical documentation [1–4]. The software allows physicians to dictate clinical notes using SRS to convert voice into electronic text, with editing in real time. Available findings suggest a range of outcomes associated with SRS use. Specifically, reduced report turnaround time has been found [5–8]. Cost-effectiveness of SRS over traditional transcription has also been noted [9]. Fewer interruptions of emergency room physicians occurred with SRS when compared to written data entry [10].

However, not all findings from SRS implementations have been positive. Some studies suggest that usability and productivity decrease with the use of SRS [11–13]. Similarly, the learning curve has been a challenge for physicians [3]. In addition, errors that arise during conversion [13] to text could potentially lead to clinical misinterpretation; quality control and feedback to users may reduce such errors over time [4, 14].

A limited number of publications on psychiatric SRS exist despite the large volume of narrative text content in mental health and addictions documentation. To date, there are two published investigations of SRS in psychiatry. One report’s findings were mixed: there were no clear benefits from a time savings, quality of care, quality of documentation or impact on workflow perspective. A limitation of this study was the small sample (n = 12) [15]. While a second study was conducted in a psychiatric setting, it did not examine physician use, as it was directed at administrative assistants and transcriptionists [16]. Thus, our objective was to further evaluate SRS in a psychiatric setting by describing psychiatrist usage and perceptions.

## Main text

The SRS evaluation was conducted using a descriptive design at the Centre for Addiction and Mental Health (CAMH) in Toronto, Canada between November 2016 and May 2017. CAMH is Canada’s largest academic mental health and addictions hospital. CAMH achieved stage 7 on the Healthcare Information Management Systems Society (HIMSS) Electronic Medical Record Adoption Model in 2017 [17]. The SRS evaluated in this paper facilitates documentation by physicians within the CAMH electronic medical record (EMR).

Specifically, the SRS used in this evaluation is Dragon Medical^©^ Network Edition 360 version 12.51.215.103 (Dragon) by Nuance. The software deployed at CAMH requires dictating into a handheld microphone or headset that is tethered to a desktop or laptop.

In October 2016 all physicians at CAMH were provided with the opportunity to participate in a limited SRS implementation. Fifty-five (n = 55) physicians indicated their interest and received a license of the Dragon Medical^©^ Network Edition 360 version 12.51.215.103, and either a Nuance PowerMic II^©^ or headset microphone. Two hours of training on Dragon Medical^©^ was provided to physicians, with additional training available as needed.

Five months after the SRS implementation, physicians received a post-implementation survey on: (1) the number of patients each physician sees per week, (2) self-reported comfort with SRS technology, (3) acceptability of the level of SRS accuracy, (4) the length of time required to complete documentation when using the SRS. Data generated by the SRS was also collected, including: (1) the number of active users, (2) average active physician user minutes, and (3) average active physician user lines. Additionally, the number of physicians who attended the additional training and the number of licenses provided were recorded.

The CAMH Research Ethics Board (REB) waived a review since we used unlinked anonymous data—and therefore deemed exempt from requiring ethical approval based on article 2.4 of the Government of Canada Tri-Council Policy Statement: Ethical Conduct for Research Involving Humans [18]. In accord, review was also waived by the CAMH Quality Projects Ethics Review (QPER) Chair.

A total of 55 SRS licenses were provided to CAMH physicians. Results of the post-implementation survey and SRS usage data are discussed below.

Fifty-three of the 55 physicians who indicated an interest in using SRS attended training, and 51 activated their SRS. Fourteen physicians attended additional optimization training by January 2017. Fifty-four physicians from the original 55 were asked to participate in a post-implementation survey, as one physician left CAMH. The post-implementation survey response rate was 38%. Respondents reported an average volume of 26.6 patients per week (range 8–80). Most reported being very comfortable (n = 12, 60%) or somewhat comfortable (n = 5, 25%) using technology. One physician reported feeling neutral, and two (10%) felt somewhat uncomfortable using technology. A majority (16/21, 76%) of physicians either somewhat agreed (8/21, 38%) or strongly agreed (8/21, 38%) that SRS reduced their time spent documenting clinical care. A majority (15/21, 71%) also either somewhat agreed (9/21, 43%) or strongly agreed (6/21, 28%) that subjectively SRS was acceptably accurate at transcribing speech.

**Fig. 1:**
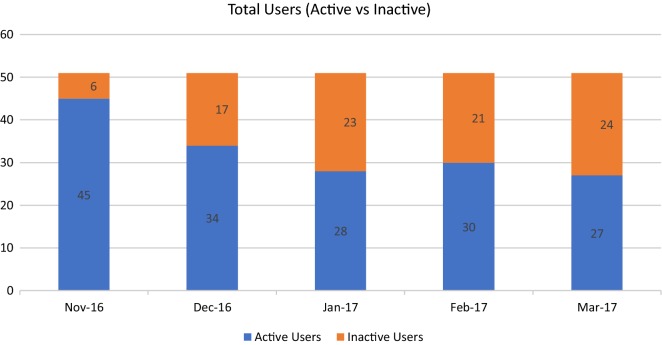
Active and inactive physician users. The number of physician users is on the y-axis. Month is on the x-axis

Data from the SRS provided the number of active users, the monthly average number of active user minutes of dictation, and the monthly average number of active user dictated lines. Figure 1 depicts total active (blue) and inactive (orange) users per month over the course of the 5-month evaluation. Most (n = 45, 88%) were active in the first month. Five months after implementation 27 physicians (53%) were still active.

**Fig. 2:**
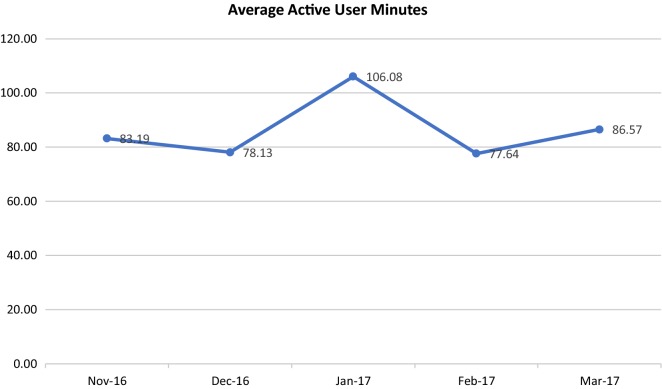
Average active physician user minutes. Average time in minutes is on the y-axis. Month is on the x-axis

**Fig. 3:**
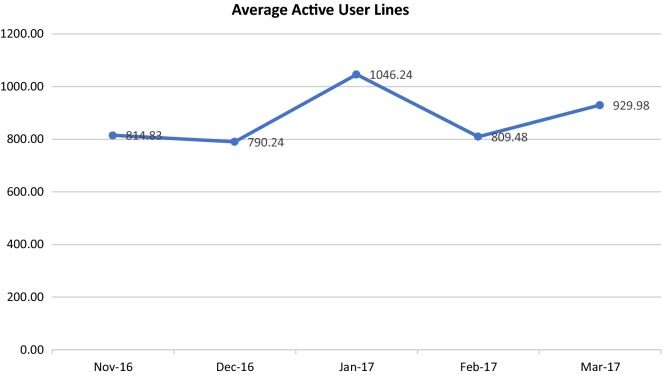
Average active physician user lines. Average number of lines is on the y-axis. Month is on the x-axis

Figures 2 and 3 show the time spent and lines generated by active users of the SRS. Specifically, Fig. 2 shows that the average number of active user minutes of dictation per month fluctuated over the period of the evaluation, with a 3.4-min increase (4%) in average active user minutes from beginning to end over the 5 months.

The average number of active user lines is seen in Fig. 3: similar to average dictated minutes, the average number of active user lines remained relatively stable, with an increase over 115 lines/month (14%) in 5 months.

## Limitations

Following initial physician enthusiasm, over the period of the 5-month evaluation, there was a 47% (24/51) drop in the number of active users. This finding is congruent with the Gartner Hype Cycle ‘trough of disillusionment’ phase, which occurs after a technology implementation [19]. Limitations to address in the future include monitoring of the number of active and inactive users over a longer period of time may provide insight into whether the remaining stages of the Gartner Hype Cycle may occur—at the time of submission the number of active licenses is stable at sixty. For future efforts, standardized assessments of satisfaction, usability and documentation quality assessments would be more informative.

Another factor contributing to the decline in active users over time may have been the voluntary nature of the SRS, and the availability of other methods available for documenting clinical notes. Physicians were not dependent on SRS—they may have opted out of SRS since CAMH physicians have been typing clinical notes for 9 years already and are generally comfortable with keyboard use. The availability of organizational transcription services is less likely to have been a factor, as CAMH transcription services are restricted to only two document types. If users experienced benefits of the SRS that were not dramatically better than the other documentation methods, they may not have wanted to put the time and effort into using SRS in their practice. In addition, it could be that some physicians never felt comfortable using the technology, and therefore discontinued their own use of it which has been an identified reason for discontinuation in the literature [3].

The results of this evaluation also suggested that the average number of active user minutes, and the average number of active user lines remained stable or slightly increased over time, with the exception of 1 month (January 2017) when a large outpatient service at CAMH increased the amount of dictation completed using SRS to catch up on a backlog new referrals. Although there was variation, the absence of decline in the active user minutes and average number of active user lines suggests that active physician users had even monthly usage. It may be that patient volumes and types of visits that lend themselves to SRS use remain relatively constant. These results differ from those of a study that reviewed the length of physician notes using SRS over time, and indicated that notes became shorter [3].

Results of the post-implementation survey indicate that most physicians reported a decreased amount of time spent documenting. There are mixed results in the literature related to time-savings with SRS [11, 13, 15]. It may be that CAMH physicians who were active users of the SRS were the main completers of the post-implementation survey, and physicians less interested in SRS may have been less likely to respond to the survey. Other limitations of this report include a lack of objective measures of satisfaction, usability, document quality, productivity and accuracy (error rates).

Finally, the results of this study add to the small body of literature on the use of SRS in a psychiatric setting. Similar to our earlier study of physician use of SRS in psychiatry, the results are mixed [15]. This may in part be a result of differences in design. The initial study used a smaller sample size, and statistical comparisons were performed. Less optimally, for the current, larger, descriptive study, no formal statistical hypothesis testing was conducted. To summarize, SRS technology may be of value to physicians in the psychiatry context. This notion is further supported by the stable number of physicians with active SRS licenses at the time of submission—since the evaluation was completed, there are now sixty active licenses. However, SRS does not appear to have universal acceptance among this unique group of physicians.

Two general observations were made by the CAMH SRS team. First, it was important to keep in regular communication with the physician users to identify any technical or education problems and address physicians’ SRS difficulties in a timely manner. Second, it takes time to learn how to effectively use the SRS and incorporate it into physician workflow. It was observed that physicians who spent time refining their use of the technology continued with SRS. However, the value proposition of SRS varies across users—some physicians gain much efficiency e.g. those who have physical challenges with typing, are slow at typing or are early technology adopters. Since 2014—well prior to our SRS implementation—most physician document types were documented by keyboard, and so many CAMH physicians gained less efficiency by already having a high comfort level with keyboard entry.

This evaluation demonstrated that SRS technology may be useful to some physicians in psychiatric settings—however, the technology is not a ‘one size fits all’ solution. Supporting physicians with post-implementation training and regular communication may help to identify challenges that physicians are having that may influence use. Future efforts should use formal assessment tools and measures. Review usage data over an extended period of time would help to identify if the Gartner Hype Cycle applies to SRS.

## Abbreviations

CAMH: Centre for Addiction and Mental Health
EMR: Electronic Medical Record
HIMSS: Healthcare Information Management Systems Society
QPER: Quality Projects Ethics Review
REB: Research Ethics Board
SRS: speech recognition software
